# MRI biomarkers and neuropsychological assessments of hippocampal and parahippocampal regions affected by ALS: A systematic review

**DOI:** 10.1111/cns.14578

**Published:** 2024-02-09

**Authors:** Sana Mohammadi, Sadegh Ghaderi, Farzad Fatehi

**Affiliations:** ^1^ Neuromuscular Research Center, Department of Neurology, Shariati Hospital Tehran University of Medical Sciences Tehran Iran; ^2^ Department of Medical Sciences, School of Medicine Iran University of Medical Sciences Tehran Iran; ^3^ Department of Neuroscience and Addiction Studies, School of Advanced Technologies in Medicine Tehran University of Medical Sciences Tehran Iran

**Keywords:** ALS, biomarkers, hippocampus, memory impairment, MRI

## Abstract

**Background and Objective:**

Amyotrophic lateral sclerosis (ALS) is a progressive motor and extra‐motor neurodegenerative disease. This systematic review aimed to examine MRI biomarkers and neuropsychological assessments of the hippocampal and parahippocampal regions in patients with ALS.

**Methods:**

A systematic review was conducted in the Scopus and PubMed databases for studies published between January 2000 and July 2023. The inclusion criteria were (1) MRI studies to assess hippocampal and parahippocampal regions in ALS patients, and (2) studies reporting neuropsychological data in patients with ALS.

**Results:**

A total of 46 studies were included. Structural MRI revealed hippocampal atrophy, especially in ALS‐FTD, involving specific subregions (CA1, dentate gyrus). Disease progression and genetic factors impacted atrophy patterns. Diffusion tensor imaging (DTI) showed increased mean diffusivity (MD), axial diffusivity (AD), radial diffusivity (RD), and decreased fractional anisotropy (FA) in the hippocampal tracts and adjacent regions, indicating loss of neuronal and white matter integrity. Functional MRI (fMRI) revealed reduced functional connectivity (FC) between the hippocampus, parahippocampus, and other regions, suggesting disrupted networks. Perfusion MRI showed hypoperfusion in parahippocampal gyri. Magnetic resonance spectroscopy (MRS) found changes in the hippocampus, indicating neuronal loss. Neuropsychological tests showed associations between poorer memory and hippocampal atrophy or connectivity changes. CA1‐2, dentate gyrus, and fimbria atrophy were correlated with worse memory.

**Conclusions:**

The hippocampus and the connected regions are involved in ALS. Hippocampal atrophy disrupted connectivity and metabolite changes correlate with cognitive and functional decline. Specific subregions can be particularly affected. The hippocampus is a potential biomarker for disease monitoring and prognosis.

## Graphical abstract

The hippocampus and connected regions are structurally and functionally altered in ALS/ALS‐FTD, as evidenced by atrophy, disrupted connectivity, metabolite changes, and hypoperfusion. Specific hippocampal subregions such as CA1, dentate gyrus, and associated white matter tracts are particularly impacted in ALS and correlate with disease progression and genetic factors.

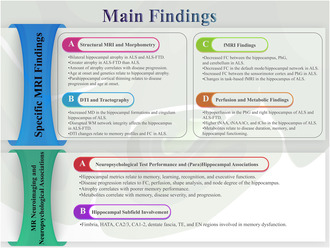

## 1 INTRODUCTION

Amyotrophic lateral sclerosis (ALS) is the most common motor neuron disease (MND) and a progressive motor and extra‐motor neurodegenerative disease.^1^ Although primarily affecting motor functions, ALS also leads to cognitive and behavioral changes, including memory impairment, executive dysfunction, emotional and learning alterations, and language deficits associated with the dysfunction of specific brain regions.^2^, ^3^ One of the specific regions involved in these functions is the hippocampal and parahippocampal regions, which play a crucial role in memory and learning processes and are affected in ALS.^4^ The hippocampal region is divided into subregions, including the dentate gyrus, cornu ammonis (CA), and the subiculum. The fascia dentata and the hilus are included in the dentate gyrus, while the CA is anatomically and functionally separated into the CA1, CA2, CA3, and CA4 subfields. The fimbria is not a subfield but a bundle of axons that connects the hippocampus to other brain regions.^5^, ^6^, ^7^, ^8^ The parahippocampal area includes the entorhinal cortex, the perirhinal cortex, and the parahippocampal gyrus (PhG).^9^

Another essential aspect of ALS is the co‐occurrence of frontotemporal dementia (FTD) in some patients.^2^, ^10^ FTD is the second most common cause of early‐onset dementia after Alzheimer's disease, and shares clinical, genetic, and pathological features with ALS.^11^, ^12^, ^13^ The term ALS‐FTD spectrum refers to various phenotypes ranging from pure ALS to pure FTD with multiple levels of motor, cognitive, and behavioral impairment.^14^, ^15^ The neuropathological link between ALS and FTD is exemplified by the presence of common protein aggregates, particularly those related to transactive response DNA‐binding protein (TARDBP).^16^ Additionally, genetic mutations have been identified as significant risk factors for ALS, with chromosome 9 open reading frame 72 (C9orf72), superoxide dismutase 1 gene (SOD1), and TARDBP representing the most common gene mutations.^17^ The C9orf72 hexanucleotide repeat expansion has been associated with both familial and sporadic ALS as well as FTD, accounting for a significant proportion of ALS‐FTD cases.^18^ Mutations in the SOD1 gene, which codes for the enzyme superoxide dismutase 1, have been related to familial ALS.^19^, ^20^ Mutations in the TARDBP gene, which codes for TDP‐43, have been detected in sporadic and familial ALS cases.^21^, ^22^ These genetic factors contribute to the heterogeneity of clinical presentations, disease progression, and cognitive dysfunction observed in ALS.^23^, ^24^

Magnetic resonance imaging (MRI) biomarker alterations provide a helpful window into understanding the progression of ALS.^25^, ^26^ MRI is a non‐invasive and multiparametric device that can measure structural and functional changes in the hippocampus and adjacent regions in ALS.^27^, ^28^ Various MRI biomarkers, such as hippocampal volumetric and quantitative measures, diffusion tensor imaging (DTI) metrics, functional connectivity (fMRI), perfusion‐weighted imaging (PWI), and magnetic resonance spectroscopy (MRS), have been used to characterize hippocampal pathology in ALS.^29^, ^30^, ^31^

Furthermore, neuropsychological assessments of memory, executive function, and behavior, including the Rey Auditory Verbal Learning Test (RAVLT), the California Verbal Learning Test (CVLT), the Rey‐Osterrieth Complex Figure Test (ROCF), the Trail Making Test (TMT), the Stroop Test, and others, are also commonly used to evaluate the cognitive and behavioral symptoms associated with hippocampal in ALS patients.^32^, ^33^, ^34^, ^35^ MRI and neuropsychological assessments are used to assess the structure and function of the hippocampal and parahippocampal regions.^36^, ^37^

The purpose of this work is to provide a comprehensive review of MRI biomarkers and neuropsychological assessments of abnormalities of the hippocampal and parahippocampal region in ALS patients in order to elucidate the role of hippocampus and parahippocampus damage in the clinical course of the disease, as well as to identify gaps and challenges for future research.

## 2 METHODS

### 2.1 Search strategy and inclusion and exclusion criteria

**FIGURE 1:**
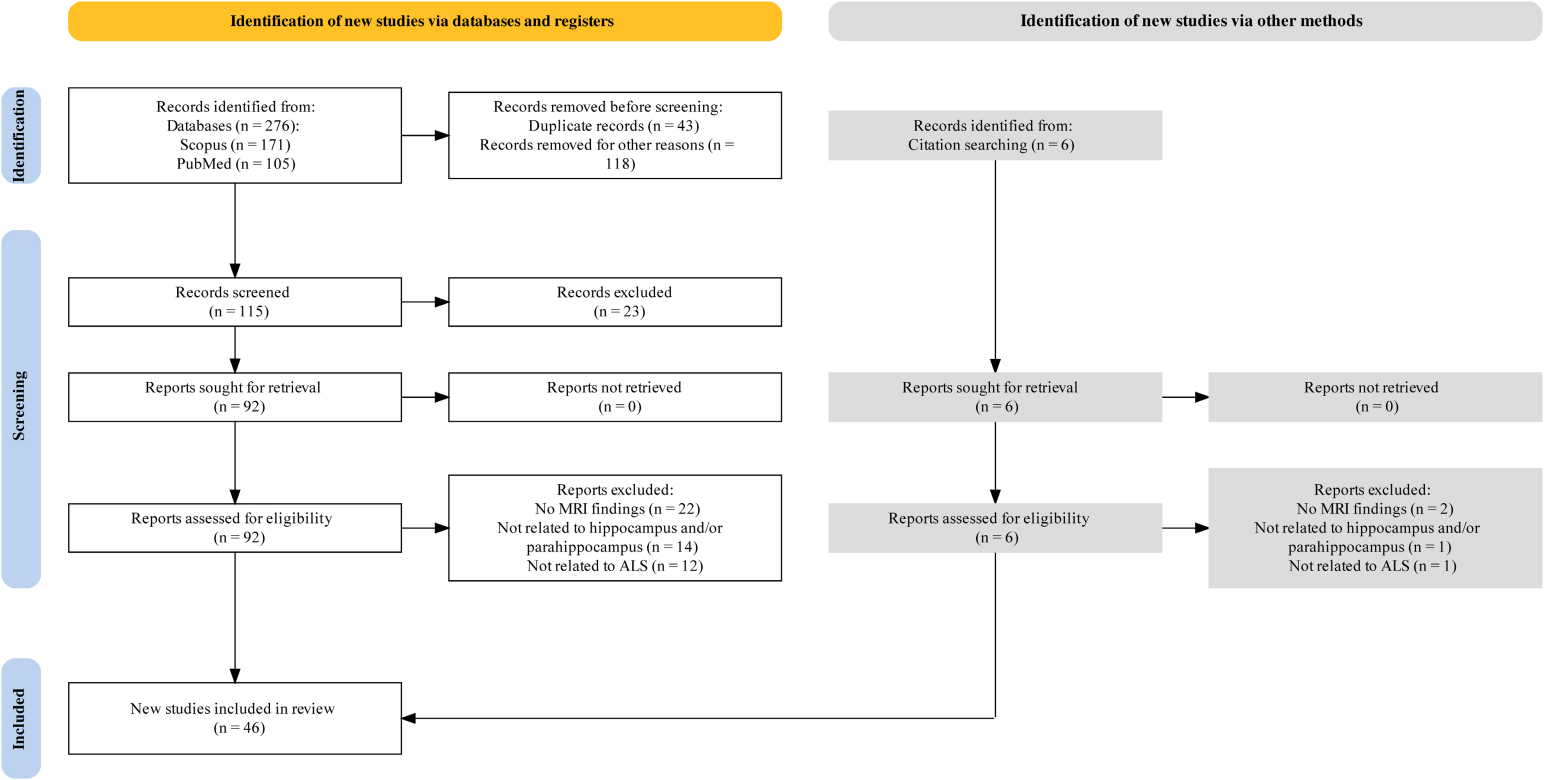
PRISMA flow diagram depicting article selection and exclusion.

**FIGURE 2:**
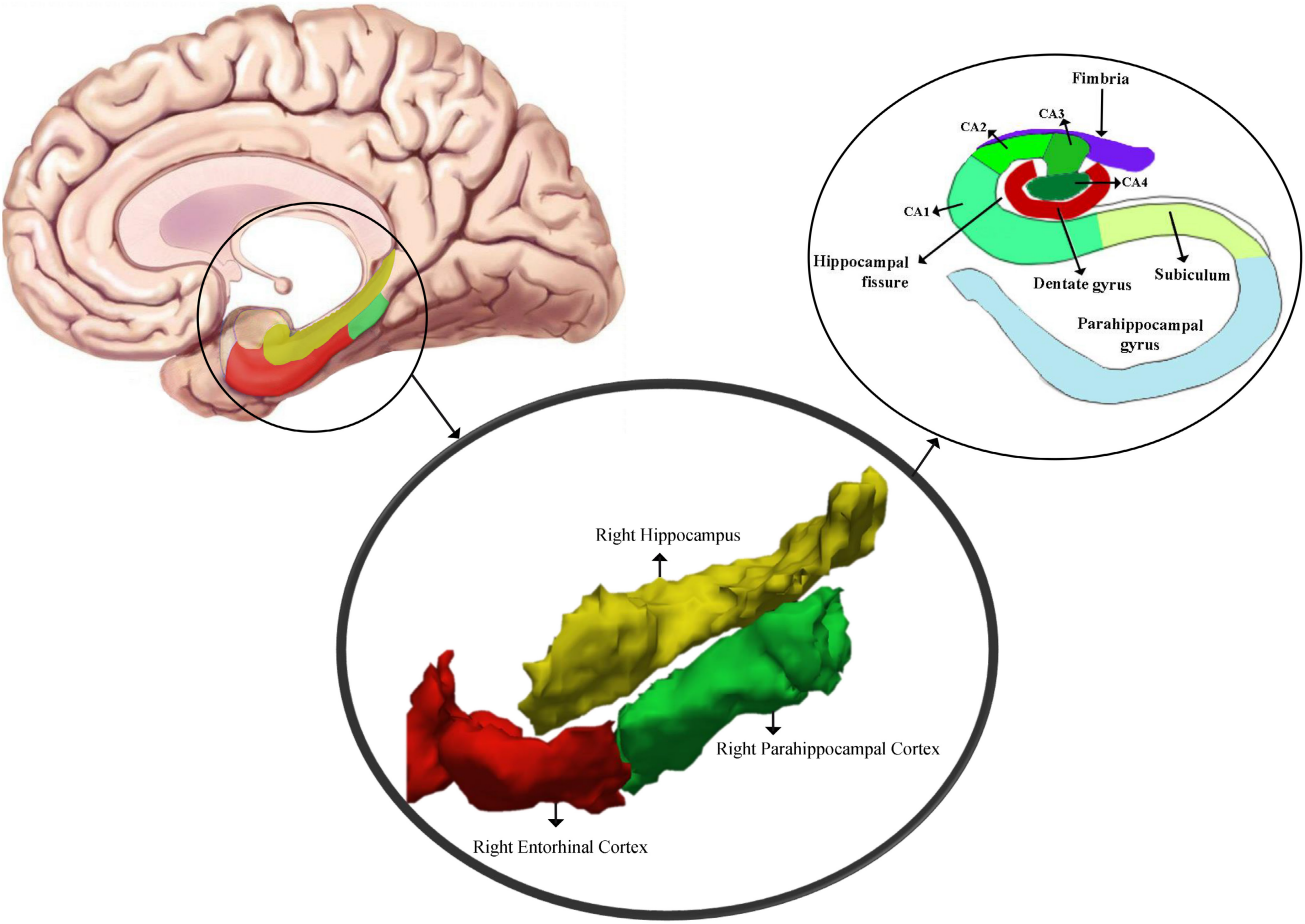
The targeted and adjacent regions examined in this systematic review (the right hippocampal, right parahippocampal cortex, and right entorhinal cortex, as analyzed using volumetric atlas‐based analysis of 3D T1‐weighted images with Freesurfer software).

This systematic review followed the Preferred Reporting Items for Systematic Reviews and Meta‐Analyses (PRISMA)^38^ standards and conducted a thorough search of the Scopus and PubMed databases (Figure 1). This systematic review was not registered in PROSPERO or any other prospective register of systematic reviews. The search strategy focused on identifying articles that studied the association between the hippocampus and adjacent regions, such as the parahippocampus, entorhinal cortex, perirhinal cortex, and parahippocampal cortex, with ALS (Figure 2). The search also included studies using MRI techniques to examine structural and functional alterations in the hippocampus and related structures in ALS patients, such as structural morphometry, fMRI, and DTI. The keywords were input as free text or MeSH phrases depending on the database. The search was limited to English items published between January 1, 2000, and July 1, 2023. For further investigation, we manually examined the reference lists of the collected articles.

Imaging studies were included, including MRI biomarker findings for hippocampal and parahippocampal regions and neuropsychological assessments associated with MRI measures of hippocampal in ALS patients. Animal studies, case reports, reviews, letters, commentary, book chapters, postmortems, and studies not written in English were excluded.

### 2.2 Data extraction and analysis methods

During data extraction, eligible studies that met the inclusion criteria were analyzed, and specific information was collected. This information included the first author and publication year, sample sizes, MRI techniques, device characteristics, and the main MRI findings summarized in Table 1. Additionally, Table 2 was used to gather and summarize the main cognitive and behavioral findings and the neuropsychological tests used in each study. The studies were then classified according to their MRI findings, neurophysiological assessment, and the associations between these two techniques. The systematic review identified several MRI biomarkers and neurophysiological assessment methods used to examine the hippocampal regions in ALS patients, including conventional, advanced, and analysis‐based metrics. Quality assessments were independently reviewed and double‐checked to ensure accuracy, and any discrepancies were resolved through discussion. The Cochrane Handbook's predefined quality assessment criteria were used to ensure that only high‐quality studies were included,^39^ which increased the credibility of the systematic review.

**TABLE 1:** Summarize MRI findings in hippocampal and parahippocampal regions.

First author	Participant (Patients/HCs)	Imaging device [Field (Tesla)/Channel Coil]	Techniques	MRI findings
Christidi (2023)^40^	12/12	3T/8	MRS	↑ Hippocampal tNAA, tNAA/tCr, and tCho bilaterally. Disease duration ↔ ↑ Right hippocampal tCho, ↓ right hippocampal Glu/tCr, and ↓ left hippocampal inositol
Rajagopalan (2022)^41^	58/14 (neurological controls)	3T/8	DTI	WM network degree using FA measure affected the frontal lobe, temporal lobe, precentral gyrus, and hippocampus in ALS‐FTD
Wang (2022)^42^	8 ALS‐FTD‐C; 6 ALS‐FTD‐M; 4 ALS‐FTD‐S/20	3T/8	T1‐w PCASL	In ALS‐FTD → ↓ GMV in hippocampus and PhG. ALS‐FTD‐M → Perfusion in the left PhG. GMV ↔ CBF in ALS‐FTD in PhG
Dieckmann (2022)^43^	100/72	1.5T/4	T1‐w	ALS vs. HC → ↓ GMV in the left hippocampus. ↓ GMV in left hippocampal CA4/dentate gyrus in higher phase patients
Ahmed (2021)^44^	41 ALS‐FTD; 52 ALS/58	3T/8	T1‐w	↓ GMV in the bilateral hippocampus in ALS‐FTD. ALS‐FTD vs. ALS → Greater atrophy in the bilateral hippocampus
Canna (2021)^45^	33/28	3T/32	T1‐w PCASL	CBF + volumes → Significant discrimination of ALS vs. HC in the right hippocampus
Ferraro (2021)^46^	29/29	1.5T/8	T1‐w	Older age at onset → ↓ GMV in frontotemporal and PhG → shorter survival
Trojsi (2021)^47^	32/21	3T/8	rs‐fMRI	ALS vs. HCs → ↓ FC between the bilateral hippocampus, PhG, and cerebellum
Ma (2021)^48^	54/54	3T/NA	rs‐fMRI	↓ d‐ReHo in left rectus gyrus and left PhG in ALS vs. HCs
Masuda (2021)^49^	30/53	3T/32	T1‐w	In ALS → ↓ Left medial orbital cortex and ↓ right hippocampus (VBM analysis)
Chen (2021)^50^	52/51	3T/64	DTI DKI NODDI	↓ RTOP in PhG of ALS patients
Liu (2021)^51^	76/94	3T/NA	T1‐w	ALS vs. HCs → ↓ Global hippocampal volumes. King's stage 2 ALS patients → ↓Left hippocampal volumes (vs. HCs). King's stage 3 ALS patients → ↓ Bilateral hippocampal volumes (vs. HCs)
Machts (2021)^52^	Longitudinal^a^: 0: 100/99 1: 79/73 2: 40/61 3: 10/16	3T/NA	T1‐w	↓ Right PhG over time in ALS vs. HCs
Chipika (2020)^53^	100/117	3T/NA	T1‐w	In ALS → Mesial temporal lobe pathology (hippocampal and amygdala)
Christidi (2019)^54^	50 ALS; 18 Alzheimer's disease/40	3T/NA	T1‐w	ALS vs. HCs and Alzheimer's disease patients ↔ ↓ Hippocampal atrophy (CA2/CA3 and HATA regions). ↓ The volume of left fimbria, bilateral hippocampal tails, right CA1, right molecular layer, and right GC‐DG in ALS‐low
Du (2019)^55^	18/16	3T/NA	DTI	↑ MD in the left cingulum bundle (hippocampal part) in ALS
Finegan (2019)^56^	100/117	3T/8	T1‐w	ALS vs. HCs → ↓ Subcortical GMV in the left hippocampus and right hippocampal atrophy. ALS vs. HCs ↔ atrophy in dentate (GC‐ML‐DG), molecular layer, ↓ HATA, CA2/3, and CA4 subfields
Bede (2018)^57^	14 C9^+^ ALS‐FTD; 12 C9^−^ ALS‐FTD; 36 ALS/50	3T/8	T1‐w	In C9^+^ ALS‐FTD → Bilateral hippocampal atrophy
Bueno (2018)^58^	20/15	3T/NA	T1‐w DTI rs‐fMRI	↓ FC in the bilateral hippocampus, PhG, and posterior cingulate in ALS. In ALS ↔ ↓ GMV in the left hippocampus, left entorhinal cortex, and right posterior cingulate. ↑ FA and ↓ MD in the left cingulum bundle (hippocampal part) in ALS vs. HCs
Ishaque (2018)^59^	Four different prospective studies 27/13 19/23 20/16 17/22	1.5T, 3T, 4.7T/NA	T1‐w	Whole‐brain analysis → Significant changes in image texture bilaterally in the motor cortex, corticospinal tract, hippocampus, etc. In short‐survivors → ↓ Autocorrelation in the bilateral motor cortex, frontal regions, insula, right hippocampus, thalamus; and texture change in the hippocampus
Machts (2018)^60^	31/29	1.5T/NA	T1‐w	ALS vs. HCs → ↓ Bilateral hippocampal volumes (especially CA1)
Menke (2018)^61^	13/NA	3T/12	T1‐w DTI	In ALS → Progressive local atrophy (including hippocampus). ↑ RD, AD, MD in hippocampus and PhG
Christidi (2017)^62^	42/25	3T/NA	T1‐w DTI	Microstructural changes in the hippocampus and frontotemporal WM tracts ↔ memory profile. ↓ Right hippocampal volume in non‐demented ALS patients. PPZ differences in ALS vs. HCs ↔ Hippocampal neuropathological changes
Buhour (2017)^63^	37/37	3T/NA	T1‐w	ALS vs. HCs → GM atrophy predominated in the temporal pole and left hippocampus
Schulthess (2016)^64^	Longitudinal: 0: 135/56 1: 27/56	NA/NA	DTI rs‐fMRI	ALS vs. HCs → ↓ FC of medial prefrontal cortex in default mode/hippocampal network. FA values ↔ FC measures in default mode/hippocampal network in ALS
Westeneng (2016)^65^	156 C9^−^ ALS; 14 C9^+^ ALS/92	3T/NA	T1‐w	C9^+^ ALS vs. C9^−^ ALS → Smaller volumes of the right hippocampus
Aho‐Özhan (2016)^66^	15/14	3T/NA	fMRI	ALS vs. HCs → ↓ Brain activity in the bilateral hippocampus while processing sad faces
Machts (2015)^67^	67 C9^−^ ALS (7 ALS‐FTD; 18 ALS‐Plus; 42 ALS‐Nci)/39	3T/32	T1‐w	ALS‐Plus vs. HCs → ↓ Bilateral hippocampal volume (especially head and body). ALS‐Plus vs. ALS‐FTD → Similar but less ↓ hippocampal volume. ↓ Hippocampus and thalamus volume → Sensitive predictors of phenotypic classification
Steinbach (2015)^68^	16/16	3T/NA	DTI	Early phase → ↓ Motor network. Late phase → Dysfunctions of hippocampus/medial temporal lobe. Parahippocampal cortex ↔ visual cortex (structural connectivity)
Zhu (2015)^69^	22/22	3T/NA	T1‐w rs‐fMRI	In ALS → ↓ GMV in bilateral precentral gyri and ↑ ALFF in right PhG
Walhout (2015)^70^	Longitudinal: 0: 112/60 1: 39/NA	3T/NA	T1‐w	↓ Left fusiform and parahippocampal cortex thickness over time in ALS vs. HCs
Westeneng (2015)^71^	Longitudinal: 0: 112/60 1: 39/NA	3T/NA	T1‐w	↓ Right CA 2/3 and 4/DG and left presubiculum volumes over time in ALS vs. HCs
Heimrath (2014)^72^	9/11	3T/NA	rs‐fMRI	ALS vs. HCs → ↑ FC in parahippocampal and parietal areas of non‐task DMN. ↑ DMN FC in parahippocampal and parietal areas → compensation for ↓ frontal networks
Abdulla (2014)^73^	58/29	3T/32	T1‐w	ALS vs. HCs → ↓ Right hippocampal volume
Stoppel (2014)^74^	26/28	3T/NA	fMRI	↓ Motor activity and ↑ novelty‐evoked hippocampal activity over time in ALS vs. HCs
Barbagallo (2014)^75^	24/22	3T/8	DTI	ALS vs. HCs → ↑ MD in the hippocampus
d'Ambrosio (2014)^76^	20/18	3T/8	T1‐w	Disease progression rate ↔ ↓ GMV in left parahippocampal cortex
Bede (2013)^30^	30 C9^−^ ALS; 9 C9^+^ ALS/44	3T/8	T1‐w DTI	C9^−^ ALS vs. HCs → ↓ Left hippocampus volume. C9^+^ ALS vs. HCs → ↓ Bilateral hippocampal volumes. Vertex‐wise shape analyses → Changes in the lateral and inferior part of the left hippocampus. ↑ AD, MD, and RD in the hippocampus of C9^+^ ALS vs C9^−^ ALS
Pettit (2013)^77^	30/30	1.5T/8	DTI	ALS vs. HCs → ↑ MD in the hippocampal portion of cingulum bundles
Agosta (2011)^78^	16 ALS with CST damage; 10 ALS with undetectable CST damage/15	1.5T/NA	DTI rs‐fMRI	Patients with no CST DTI abnormalities → ↑ FC to left SMC (more than the whole group). ALS vs. controls → ↑ FC between left SMC and PhG. In ALS → ↑ FC between right SMC and right PhG
Senda (2011)^79^	Longitudinal: 0: 17/17 1: 17/NA	3T/NA	DTI	↑ MD in hippocampal bilaterally
Thivard (2007)^80^	15/25	1.5T/NA	T1‐w DTI	ALS vs. HCs → ↓ GMV and ↑ MD in hippocampal bilaterally

Abbreviations: AD, axial diffusivity; ALFF, amplitude of low‐frequency fluctuations; ALS, amyotrophic lateral sclerosis; ALS‐FTD, ALS‐frontotemporal degeneration; ALS‐FTD‐C, cognitive‐onset ALS‐FTD; ALS‐FTD‐M, motor‐onset ALS‐FTD; ALS‐FTD‐S, simultaneous‐onset ALS‐FTD; ALS‐high, ALS‐high memory performance; ALS‐low, ALS‐low memory performance; ALS‐Nci, no cognitive impairment; ALS‐Plus, cognitive and/or behavioral impairment; C9^−^, patients without C9orf72 hexanucleotide expansions; C9^+^, patients with C9orf72 hexanucleotide expansions; CA, Cornu Ammonis; CBF, cerebral blood flow; CST, corticospinal tract; DKI, diffusion Kurtosis imaging; DMN, default mode network; d‐ReHo, dynamic regional homogeneity; DTI, diffusion tensor imaging; EN, entorhinal region; FA, fractional anisotropy; FC, functional connectivity; GC‐DG, granule cell layer of the dentate gyrus; GC‐ML‐DG, granule cell layer of the molecular layer of the dentate gyrus; Glu, glutamate; GMV, gray matter volume; HATA, Hippocampal‐amygdala transition area; HCs, healthy controls; MD, mean diffusivity; MRS, magnetic resonance spectroscopy; NA, not applicable; NODDI, neurite orientation dispersion and density imaging; PCASL, Pseudo‐continuous arterial spin labeling; PhG, parahippocampal gyrus; PPZ, perforant pathway zone; RD, radial diffusivity; rD50 < 0.25, phase I; 0.25 ≤ rD50 < 0.50, phase II; rD50 ≥ 0.50, phases III/IV; rD50, relative disease aggressiveness; rs‐fMRI, resting‐state functional magnetic resonance imaging; RTOP, return‐to‐origin probability; SMC, sensorimotor cortex; T1‐w, T1‐weighted; tCho, total choline; tCr, total creatine; TE, transentorhinal region; tNAA, total N‐acetylaspartate; VBM, voxel‐based morphometry; WM, white matter. Symbols mean: ↑: “increase” or “elevated”; ↓: “decrease” or “reduced”; ← or →: “leads to” or “results in”; ↓↑: “fluctuation” or “variation”; ↔: “bidirectional”, “correlation”, “contribution”, “associated with”, “two‐way”, and “correlate to or with”.

^a^ In longitudinal studies, 0: first phase, 1: second phase, 2: third phase, 3: fourth phase.

**TABLE 2:** MR neuroimaging and neuropsychological findings in hippocampal and parahippocampal regions.

First author	Participant patients/HCs	Neuropsychological test	MRI findings cognitive and clinical assessment
Christidi (2023)^40^	12/12	ECAS	Superior memory ↔ ↑ Bilateral hippocampal tNAA/tCr. Higher ALSFRS‐r ↔ ↓ tCho and ↑ tNAA/tCr
Rajagopalan (2022)^41^	58/14 (neurological controls)	MoCA	Disease progression rate in ALS ↔ Node degree of ALS‐FTD in right angular gyrus (negative correlations) and right hippocampus (positive correlations)
Dieckmann (2022)^43^	100/72	ECAS MMSE	Analyses with rD50 → ↓ Hippocampal volume bilaterally
Ahmed (2021)^44^	41 ALS‐FTD; 52 ALS/58	ACE‐III	↑ ACE‐III Total scores ↔ ↑ Hippocampus volume. Memory difficulties ↔ ↓ Hippocampus volume
Machts (2021)^52^	Longitudinal^a^: 0: 100/99 1: 79/73 2: 40/61 3: 10/16	RAVLT TMT ST	Memory, learning, recall, and recognition impairment ↔ ↓ Bilateral hippocampal volume over time. Left PhG thinning ↔ Poorer learning performance
Tae (2020)^81^	32/43	ALSFRS	Delta ALSFRS scores ↔ ↓ Right hippocampus shape distances
Christidi (2019)^54^	50; 18 Alzheimer's disease/40	BSRT	ALS‐Low vs. ALS‐High → ↓ Right hippocampus volume and shape differences in the lateral aspect of the left hippocampus. ALS‐High vs. ALS‐Low → ↓ Left hippocampus lateral shape. ALS‐Low vs. Alzheimer's disease → Higher hippocampus volume bilaterally. ALS‐Low → Fimbria and HATA atrophy. ALS‐High → HATA and CA2/3 atrophy
Lulé (2018)^82^	139/NA	ECAS	Memory impairment → Anatomical changes in hippocampal regions. TE and EN involvement in ALS ↔ CA1/2 and dentate fascia atrophy ↔ Memory dysfunction
Benbrika (2018)^83^	28/30	HSCT LNS VF TMT DIF	Total and DIF ↔ ↓ Prefrontal cortex, right superior temporal pole, and PhG volume. ALS vs. HCs → ↑ Total and DIF of TAS‐20 → ↑ Alexithymia in patients
Buhour (2017)^63^	37/37	TMT VF	Episodic memory (immediate recall) ↔ ↓ Metabolic value of bilateral hippocampus and left PhG. Delayed recall ↔ ↓ Metabolic value of left PhG
Schulthess (2016)^64^	Longitudinal: 0: 135/56 1: 27/56	ALSFRS‐r	ALSFRS‐r score ↔ ↓ FC in default mode/hippocampal network in ALS over time
Machts (2015)^67^	67 C9^−^ ALS (7 ALS‐FTD; 18 ALS‐Plus; 42 ALS‐Nci)/39	Letter fluency and flexibility Semantic fluency and flexibility TMT ST BDS FrSBe	Hippocampal atrophy ↔ Verbal memory performance
Zhu (2015)^69^	22/22	MMSE MoCA SCWT WCST FAB	↑ ALFF in right PhG ↔ ↑ ALS progression rate. ↓ Cognitive and executive performance in ALS ↔ ALFF in parahippocampal cortices
Raaphorst (2015)^84^	26/21	RAVLT RBMT Doors Test A Doors Test B	Hippocampal GMV ↔ Prose memory impairment in ALS without FTD
Abdulla (2014)^73^	58/29	MoCA VF TMT RAVLT RCFT	Verbal memory impairment ↔ Left hippocampal volume in ALS
Stoppel (2014)^74^	26/28	VLMT VALT RCFT FFT TMT VF	ALSFRS‐r ↔ Motor activity decline (positive correlations), and hippocampal activation increase (negative correlations)
Barbagallo (2014)^75^	24/22	MMSE BDI‐II RAVLT COWAT MCST FAB	MD measures ↔ MCST and FAB scores of ALS patients (negative correlation) and neuropsychological dysfunctions (positive correlation)
Agosta (2011)^78^	16 ALS with CST damage; 10 ALS with undetectable CST damage/15	ALSFRS‐r	ALSFRS‐r ↔ ↑ FC between left SMC and right PhG

Abbreviations: ACE‐III, third edition of the Addenbrooke's cognitive examination; ALFF, amplitude of low‐frequency fluctuations; ALS, amyotrophic lateral sclerosis; ALSFRS‐r, amyotrophic lateral sclerosis functional rating scale‐revised; ALS‐FTD, ALS‐frontotemporal degeneration; ALS‐high, ALS‐high memory performance; ALS‐low, ALS‐low memory performance; ALS‐Nci, no cognitive impairment; ALS‐Plus, cognitive and/or behavioral impairment; BDI, beck depression inventory; BDI‐II, beck depression inventory‐II; BDS, backward digit span; BSRT, Babcock story recall test; C9^−^, patients without C9orf72 hexanucleotide expansions; C9^+^, patients with C9orf72 hexanucleotide expansions; CA, cornu ammonis; COWAT, controlled oral word association test; CST, corticospinal tract; DIF, difficulty identifying feelings scores; ECAS, Edinburgh cognitive and behavioral ALS screen; EN, entorhinal region; FAB, frontal assessment battery; FC, functional connectivity; FFT, figural fluency test; FrSBe, frontal systems behavior scale; GMV, gray matter volume; HATA, Hippocampal‐amygdala transition area; HCs, healthy controls; HSCT, Hayling sentence completion test; LNS, letter‐number sequencing task; MCST, modified card sorting test; MD, mean diffusivity; MMSE, mini‐mental state examination; MoCA, montreal cognitive assessment; NA, not applicable; PhG, parahippocampal gyrus; RAVLT, Rey auditory verbal learning test; RBMT, Rivermead Behavioral Memory Test; RCFT, Rey complex figure test; rD50, relative disease aggressiveness; SCWT, Stroop color‐word interference test; SMC, Sensorimotor cortex; ST, Stroop test; TAS‐20, Toronto Alexithymia Scale; tCho, total choline; tCr, total creatine; TE, transentorhinal region; TMT, trail making test; tNAA, total N‐acetylaspartate; VALT, visual associate learning test; VF, verbal fluency task; VLMT, verbal learning and memory test; WCST, Wisconsin card sorting test; WM, white matter. Symbols mean: ↑: “increase” or “elevated”; ↓: “decrease” or “reduced”; ← or →: “leads to” or “results in”; ↓↑: “fluctuation” or “variation”; ↔: “bidirectional”, “correlation”, “contribution”, “associated with”, “two‐way”, and “correlate to or with”.

^a^ In longitudinal studies, 0: first phase, 1: second phase, 2: third phase, 3: fourth phase.

## 3 RESULTS

### 3.1 Overview of results

Our results provide an overview of the results of various studies that utilized different MR neuroimaging techniques to investigate MRI biomarkers and neuropsychological evaluation of hippocampal regions affected by ALS and ALS‐FTD compared to healthy controls (HCs) or other control groups. In summary, 46 studies were eligible for additional evaluation.

**FIGURE 3:**
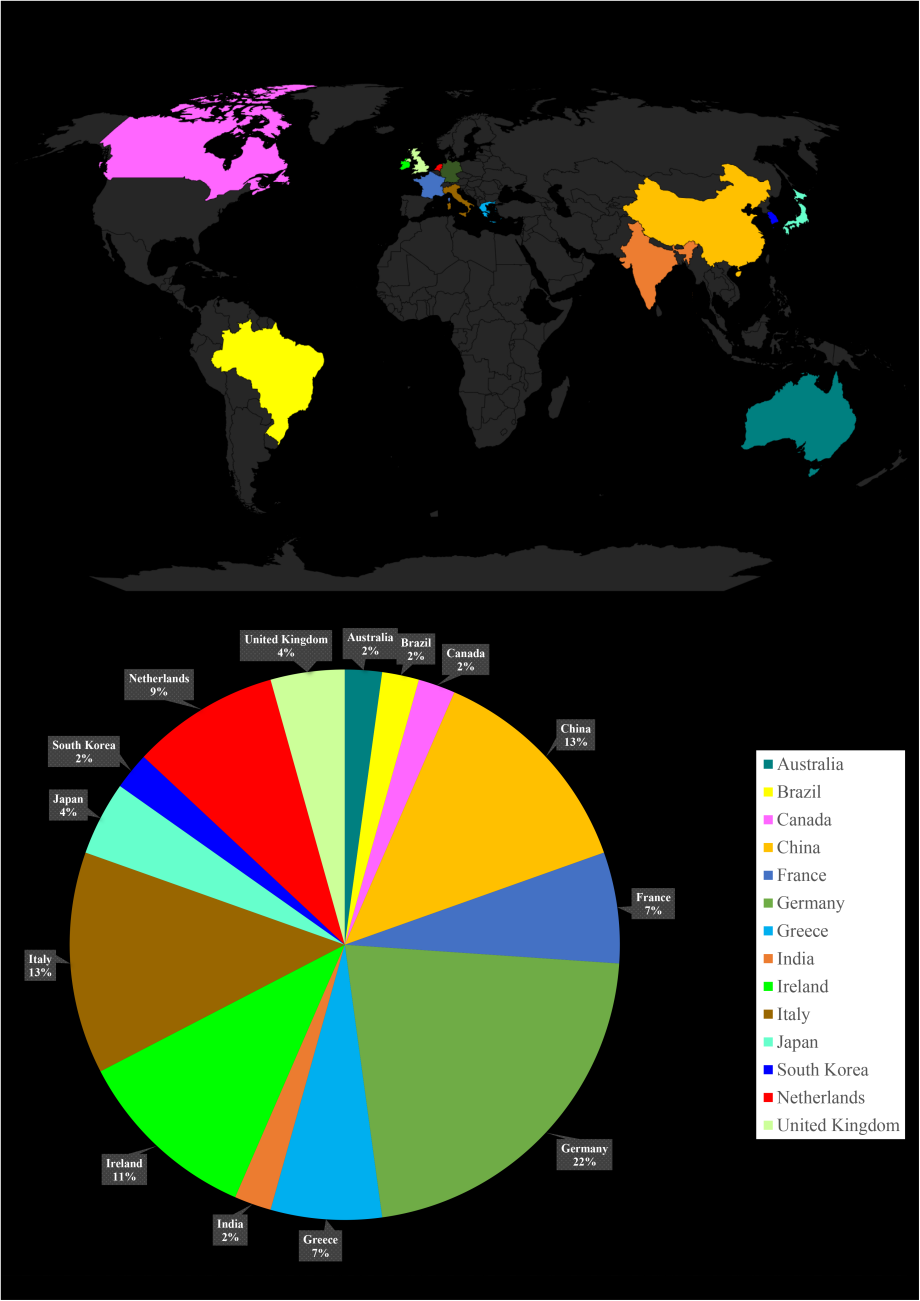
Global distribution of countries contributing to the systematic review.

Our systematic review analyzed the geographical distribution of studies on MRI biomarkers and neuropsychological assessments in ALS based on the first author's affiliation (Figure 3). The analysis revealed uneven global representation, with most of the contributions coming from selected countries. Specifically, Germany (*n* = 10), China (*n* = 6), Italy (*n* = 6), Ireland (*n* = 5), and the Netherlands (*n* = 4) collectively accounted for approximately 67% of the included articles. European nations exhibited the highest participation rate, comprising 71.7% (33 out of the 46) of studies. Prominent contributions were from Germany, Italy, Ireland, the Netherlands, Greece (*n* = 3), France (*n* = 3), and the United Kingdom (*n* = 2). The remaining countries contributed 1–2 articles each, including Japan, Australia, Brazil, Canada, India, and South Korea. Given the geographical concentration observed, we recommend future efforts to improve population heterogeneity through targeted recruitment across underrepresented world regions. Expanding diagnostic research globally will improve the generalizability of systematic reviews and provide a more comprehensive understanding of ALS epidemiology, particularly regarding genetic diversity. Moreover, investigating diverse populations and countries may reveal previously undiscovered disease characteristics. This geographical distribution analysis highlights the need for broader international representation in ALS imaging and neuropsychology research associated with hippocampal and parahippocampal regions and related cognitive‐behavioral impairments.

MRI‐based biomarker findings in hippocampal and parahippocampal regions are summarized in Table 1. Studies were carried out between 2007 and 2023. The different MR neuroimaging techniques used in these studies include T1‐weighted (T1‐w) imaging, pseudo‐continuous arterial spin labeling (PCASL), MRS, DTI, and resting‐state fMRI (rs‐fMRI). Most studies employed 3T MRI scanners, with a few using 1.5T and 4.7T devices. Most studies used 8‐channel head coils; some used 4, 12, 32, or 64‐channel coils, and others did not report this information. The studies covered a wide range of participant populations, including patients with different ALS and FTD subtypes and those with genetic mutations such as C9orf72 expansions (C9^+^). Longitudinal studies were also conducted, with follow‐up time points. In terms of techniques, T1‐w was the most commonly employed method, used either alone (*n* = 19) or in combination with other techniques like DTI (*n* = 4), PCASL (*n* = 2), and fMRI (*n* = 1). DTI was also used frequently (*n* = 7), followed by fMRI (*n* = 5). MRS was less commonly used (*n* = 1).

Table 2, included in this review, utilizes a range of neuropsychological tests to assess cognitive and/or behavioral impairments in patients with ALS. Some of the tests that were used most frequently in these studies were the Edinburgh Cognitive and Behavioral ALS Screen (ECAS), the Montreal Cognitive Assessment (MoCA), the TMT, and the RAVLT. Other tests used in multiple studies include the Mini‐Mental State Examination (MMSE), the Frontal Assessment Battery (FAB), and the Verbal Fluency Task (VF). These assessments targeted various cognitive domains such as memory, executive function, language, attention, and behavioral aspects.

### 3.2 Specific MRI findings

#### 3.2.1 Structural MRI and morphometry

Several studies reported bilateral hippocampal atrophy,^30^, ^42^, ^44^, ^51^, ^53^, ^57^, ^59^, ^67^, ^80^ while others observed atrophy in specific regions of the left^30^, ^43^, ^56^, ^63^, ^73^ or the right hippocampus.^45^, ^49^, ^56^, ^62^, ^65^, ^73^ In some cases, volume reductions were limited to particular subfields, such as CA4/dentate gyrus,^43^, ^56^, ^70^ CA2/CA3,^54^, ^56^, ^71^ and CA1.^60^

Bede et al.^30^ reported that the difference between C9^+^ ALS and C9^−^ ALS in hippocampal volume loss is bilateral or unilateral volume loss, while Westeneng et al.^65^ focused on the amount of volume loss and reported that C9^+^ ALS patients had greater volume loss in the right hippocampus compared to C9^−^ ALS. Furthermore, one study reported bilateral hippocampal volume loss in C9^+^ ALS‐FTD and observed subcortical GM atrophy in C9^+^ ALS‐FTD patients, limited to the bilateral thalami, hippocampi, and right accumbens nucleus.

Bilateral hippocampal atrophy in ALS‐FTD has been reported,^42^, ^44^, ^67^ and the amount of atrophy is more significant than in other ALS phenotypes, such as ALS‐Plus or ALS.^44^, ^67^ One study conducted by Machts et al.^67^ reported that ALS‐Plus showed significant bilateral hippocampal atrophy compared to HCs, especially in the head and body of the hippocampal. In another study, shape analysis of subcortical structures revealed progressive local atrophy, including the hippocampus.^61^

The volume reduction in multiple parts of the hippocampus in different stages of the disease differs, but the most significant aspect is the more considerable decrease in volume and shifting into bilateral involvement of the hippocampus in the more advanced stages.^43^, ^51^ In line with previous studies, Christidi et al.^54^ found that ALS patients with worse memory had a specific pattern of hippocampal atrophy in the left fimbria, both hippocampal tails, the right CA1, the right molecular layer, and the right GC‐DG.

Age at the symptom onset and genetic factors were also related to hippocampal atrophy. Ferraro et al.^46^ found that older age at the time of symptom onset was associated with greater frontotemporal cortical thinning, including parahippocampal cortices, while Ishaque et al.^59^ reported that shorter survival in ALS patients was related to changes in the hippocampus and other extra‐motor regions.

VBM analysis of the parahippocampal regions revealed bilateral thinning and loss of GM^46^, ^70^ or unilaterally,^52^, ^76^ and this alteration can be associated with disease progression,^76^ older age at the onset of symptom,^46^ and progress over time.^52^, ^70^

#### 3.2.2 DTI and tractography

Regarding DTI biomarkers, five studies found an increased MD in the hippocampal formations of ALS patients compared to controls, especially in the cingulum bundle (hippocampal part),^55^, ^75^, ^77^, ^79^, ^80^ while Bueno et al.^58^ showed an increased fractional anisotropy (FA) and decreased MD in the left cingulum bundle (hippocampal part) of ALS patients. Two studies demonstrated increased RD, AD, and MD in the hippocampus and parahippocampal regions in ALS patients^61^ and in the hippocampus of C9^+^ ALS patients compared to C9^−^ ALS patients.^31^ According to Rajagopalan et al.,^41^ the integrity of the WM network measured by FA was disrupted in patients with ALS‐FTD, affecting the frontal lobe, temporal lobe, precentral gyrus, and hippocampus.

The highlight of the association between DTI findings and other biomarkers is crucial. In ALS patients, Schulthess et al.^64^ showed significant correlations between FA values and FC measures within the default mode/hippocampal network. Christidi et al.^62^ found microstructural changes in the hippocampus and frontotemporal WM pathways, resulting in a unique impact on the memory profiles of ALS patients.

Finally, further analysis of diffusion data on proton position showed decreased return‐to‐origin probability (RTOP) in the PhG of ALS patients.^50^

#### 3.2.3 fMRI findings

fMRI data revealed a decrease in FC between the bilateral hippocampus, the bilateral parahippocampal gyri, and the cerebellum in ALS patients compared to HCs.^47^ Similarly, another study reported decreased FC in the bilateral hippocampus, bilateral anterior and posterior PhG, and posterior cingulate in ALS patients.^58^ Additionally, Ma et al.^48^ found a lower d‐ReHo in the left rectus gyrus and the left PhG in patients with ALS compared to HCs.

Schulthess et al.^64^ observed significantly decreased FC of the medial prefrontal cortex, a major node within the default mode/hippocampal network, in ALS patients compared to HCs. Furthermore, patterns of increased FC were observed in the analysis of the default mode/hippocampal network in ALS patients. Increased FC was observed in parahippocampal and parietal areas of the non‐task‐associated DMN,^72^ between the left sensorimotor cortex (SMC) and the right PhG,^78^ between the right SMC and the right PhG, and between the right SMC and the right PhG.^78^ Zhu et al.^69^ identified increased ALFF values in the right PhG in the sporadic ALS group.

In two studies using task‐based fMRI, one reported significant differences between ALS patients and HCs in response to sad facial expressions, with reduced brain activity observed in the hippocampus bilaterally for the ALS patients.^66^ Another reported that novelty‐evoked hippocampal activity increased across 3 months in ALS patients, possibly reflecting the build‐up of compensatory processes typically observed at the beginning of lesions. Motor activity, in contrast, decreased during the same interval.^74^

#### 3.2.4 Perfusion and metabolic findings

The pCASL MRI technique to investigate hippocampal regions affected by ALS and to differentiate ALS patients from HCs was used in two studies.^42^, ^45^ ROC analysis of cerebral blood flow (CBF) demonstrated a significant discrimination of ALS patients from HCs in the right hippocampus.^45^ On the other hand, hypoperfusion in ALS‐FTD‐M is limited to the left PhG.^42^

Using the MRS technique, Christidi et al.^40^ reported several findings related to the hippocampal regions affected by ALS. The study observed a higher bilaterality of hippocampal tNAA, tNAA/tCr, and tCho bilaterally. Additionally, disease duration was positively associated with right hippocampal tCho and negatively related to right hippocampal Glu/tCr and left hippocampal inositol.

### 3.3 MR neuroimaging and neuropsychological associations

#### 3.3.1 Neuropsychological test performance and (para)hippocampal associations

Several studies have reported associations between neuropsychological test performance and hippocampal region metrics in patients with ALS. Christidi et al.^40^ found that superior memory performance on the ECAS was associated with higher hippocampal tNAA/tCr bilaterally. Similarly, Ahmed et al.^44^ reported that the hippocampus volume was positively correlated with higher ACE‐III total scores, and memory difficulties were negatively correlated with the volume of some areas, including the hippocampus. There are significant negative correlations between episodic memory and the metabolic value of the bilateral hippocampus and left PhG.^63^ In addition, negative correlations between delayed recall and metabolic values of the left PhG were reported.^63^ Bilateral hippocampal atrophy and anatomical changes were associated with learning, recall, recognition,^52^ and memory impairment,^82^ respectively. Also, left PhG thinning was associated with poorer learning performance.^52^ Increased alexithymia (based on the higher total score and DIF sub‐score of the TAS‐20) in ALS patients was associated with significantly and negatively correlated GMV of the prefrontal cortex, right superior temporal pole, and PhG.^83^

In terms of disease progression, a positive correlation between ALSFRS‐r and increased FC was reported between the left primary SMC and the right PhG and cerebellum^78^ and a negative correlation between ALSFRS‐r and higher hippocampal activation,^74^ and delta ALSFRS negatively correlated with local shape distances in the right hippocampus.^81^ Also, higher ALSFRS‐r was associated with lower hippocampal tCho and higher tNAA/tCr.^40^

Some other studies focused on disease progression were conducted. Strong correlations were found between disease progression rate and degree of node degree in the right angular gyrus and hippocampus of ALS‐FTD patients.^41^ The correlations were negative in the right angular gyrus and positive in the right hippocampus.^41^ Another study reported that the ALS progression rate was positively correlated with the increased ALFF value in the right PhG.^69^ Ultimately, Dieckmann et al.^43^ found that decreasing bilateral hippocampal volume was associated with the parameter relative disease aggressiveness (rD50).

Additionally, several studies found correlations between hippocampal atrophy and memory performance in ALS patients.^67^, ^73^, ^84^ ALS patients exhibited poor performance on neuropsychological tests (cognitive and executive tests) that correlated with the ALFF values in the PhG.^69^ Furthermore, the correlations between neuropsychological test scores (MCST and FAB scores) and MD measures in the hippocampus highlight the role of the hippocampus in cognitive dysfunction in ALS patients.^75^

#### 3.3.2 Hippocampal subfield involvement

Fimbria and HATA were particularly atrophic in the ALS‐Low memory performance group, while HATA and CA2/3 were the most affected subfields in the ALS‐High memory performance group.^54^ The contrast between the neuropsychologically defined ALS‐High and ALS‐Low groups also revealed significant shape differences in the lateral aspect of the left hippocampus.^54^ The CA1‐2 hippocampal areas and dentate fascia, as well as transentorhinal region (TE) and entorhinal region (EN) regions, were associated with memory dysfunction in ALS patients.^82^

## 4 DISCUSSION

**FIGURE 4:**
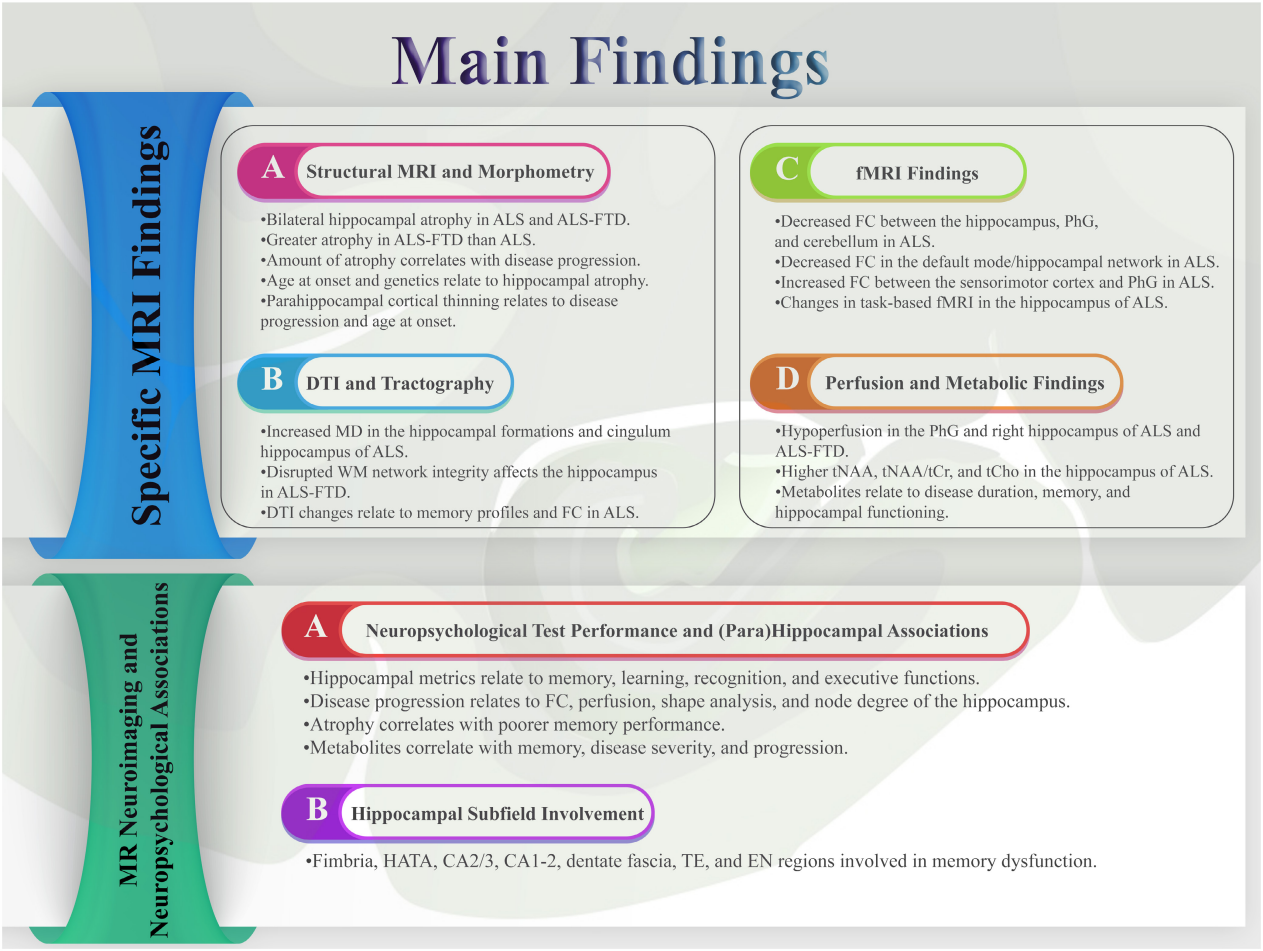
Primary MRI and neuropsychological findings of hippocampal and parahippocampal regions in ALS patients.

The objective of the current study was to examine MRI biomarkers and neuropsychological evaluations of the impact of ALS on the hippocampal and parahippocampal regions (Figure 4).

The findings of this systematic review indicate that hippocampal and parahippocampal atrophy is a common feature of ALS,^30^, ^42^, ^43^, ^44^, ^45^, ^46^, ^49^, ^51^, ^52^, ^54^, ^56^, ^57^, ^58^, ^60^, ^61^, ^62^, ^63^, ^65^, ^67^, ^69^, ^70^, ^71^, ^73^, ^76^, ^80^ with varying degrees of severity and distribution across studies. The observed atrophy patterns were more prominent in ALS‐FTD patients,^42^, ^57^, ^67^ with the bilateral hippocampus being consistently affected.^30^, ^42^, ^44^, ^46^, ^51^, ^54^, ^56^, ^57^, ^60^, ^67^, ^69^, ^70^, ^80^ Several studies reported atrophy in specific hippocampal subregions, such as CA1, CA2/CA3, CA4/DG, and HATA,^30^, ^43^, ^54^, ^56^, ^60^, ^71^ highlighting the potential for these regions as biomarkers for disease progression and severity. As a result, whereas hippocampus reduction is a typical characteristic of ALS, the link between atrophy of the hippocampus and disease progression remains unknown.

Hippocampal and parahippocampal regions' involvement in ALS appears to be dynamic, with progressive local atrophy observed in some studies and correlations between disease progression rate and GM loss in specific regions of these areas.^52^, ^70^, ^76^ Additionally, the C9^+^ ALS‐FTD was associated with more extensive hippocampal atrophy,^57^ suggesting a genotype–phenotype relationship. According to recent research, the loss‐of‐function impact of C9orf72, combined with certain gain‐of‐function entities, is required to develop a severe FTD/ALS phenotype.^85^ TDP‐43 is a protein involved in RNA metabolism linked to the development of ALS and FTD.^86^ Alzheimer's disease can occasionally result in neuronal death and gliosis in the hippocampus, a kind of TDP‐43 pathology known as hippocampal sclerosis.^87^ According to research, limbic‐predominant age‐related TDP‐43 encephalopathy (LATE) is associated with a progressive amnestic state that resembles Alzheimer's symptoms.^88^

Furthermore, LATE is a newly identified dementia that impairs memory and reasoning, such as Alzheimer's disease, but with distinct underlying reasons.^88^, ^89^ Aberrant TDP‐43 protein clusters cause LATE, which is also implicated in other neurological disorders such as ALS and FTD.^88^ Hippocampus atrophy in cases with LATE neuropathological change (NC) is more extensive than in patients with pure Alzheimer's disease, with stronger connections between hippocampal atrophy and LATE‐NC with hippocampal sclerosis pathology.^90^, ^91^ LATE is a newly suggested mention of TDP‐43 proteinopathy, which mainly affects the older medial temporal lobe.^92^ According to a recent molecular study, the amygdala and hippocampus are vulnerable to TDP‐43 disease in elderly ALS patients.^93^ As a result, it seems that TDP‐43 is linked to hippocampus atrophy in ALS patients. However, further studies on the LATE‐NC characteristics in ALS and ALS/FTD patients are required, particularly using MRI.

The peak age of onset for ALS is between 55 and 70 years, with a male predominance.^94^ The study highlighted the potential influence of age at symptom onset^46^ and genetic factors on hippocampal atrophy in ALS patients.^30^, ^57^, ^65^ These findings suggest that different disease mechanisms can underlie the observed atrophy patterns. Further research is needed to elucidate the relationship between genetic factors, age at onset, and hippocampal involvement in ALS.

Approximately, 50% of ALS patients develop cognitive impairment throughout the disease^95^ (p. 62). Worse memory performance in ALS patients was associated with volume reductions in various hippocampal subregions,^54^ highlighting the relationship between hippocampal atrophy and cognitive decline. Some studies have shown that ALS is characterized by global volume loss and local atrophy in the CA1 area of the hippocampus,^54^, ^60^ which can serve as a neural correlate for the cognitive and behavioral deficits associated with ALS. The association between hippocampal atrophy and cognitive decline in patients with ALS underscores the importance of evaluating cognitive function in clinical settings, as hippocampal atrophy can help identify patients at risk for cognitive decline or dementia. Hippocampal atrophy in patients with shorter survival suggests it can also have prognostic value in ALS.^59^

Several studies have reported significant associations between metrics from the hippocampal region, including volume, metabolic values, FC, measures from the hippocampal subfield, and memory performance in ALS patients.^40^, ^41^, ^43^, ^44^, ^52^, ^54^, ^63^, ^64^, ^67^, ^69^, ^73^, ^74^, ^75^, ^78^, ^81^, ^82^, ^83^, ^84^ The hippocampus is essential in episodic memory, learning, and recall.^96^, ^97^ Atrophy and anatomical changes in the hippocampus are linked to memory impairment in ALS.^44^, ^52^, ^54^, ^67^, ^73^, ^81^, ^82^, ^83^, ^84^ Also, the PhG, which is functionally and anatomically connected to the hippocampus, shows correlations with memory performance.^52^, ^63^, ^82^, ^83^

Regarding disease progression, the studies found mixed results.^40^, ^41^, ^43^, ^63^, ^64^, ^74^, ^78^, ^81^ Some reported a positive association between ALSFRS‐r and hippocampal volume or functional connectivity, while others found a negative correlation. The discrepancies could be due to methodological differences, sample size, disease duration, or other factors. However, the overall findings suggest the involvement of the hippocampal region in ALS progression. The hippocampus could be a biomarker to monitor disease progression and predict prognosis in ALS patients.

The studies that evaluated hippocampal subfields reported that areas like CA1‐2, dentate fascia, fimbria, and HATA were particularly affected in ALS patients with memory impairment.^54^, ^82^ The lateral aspect of the left hippocampus also showed significant shape differences between ALS patients with high and low memory performance.^54^ Furthermore, subfield analysis can provide more information about hippocampal involvement in ALS patients with cognitive and memory dysfunction.

The findings of this systematic review highlight the involvement of the hippocampal and parahippocampal regions in ALS patients, as evidenced by various DTI measures.^30^, ^41^, ^50^, ^55^, ^58^, ^61^, ^62^, ^64^, ^68^, ^75^, ^77^, ^78^, ^79^, ^80^ Most studies demonstrated increased MD in ALS patients compared to controls, which can suggest a loss of neuronal integrity in these regions. This finding was reported in seven studies,^55^, ^61^, ^62^, ^75^, ^77^, ^78^, ^79^, ^80^ and it involved both the hippocampal GM and the WM tracts connected to it, such as the cingulum bundle and the PhG. Additionally, FA and other diffusivity measures (RD and AD) were found to be altered in the hippocampal and parahippocampal regions,^30^, ^41^, ^58^, ^61^ further emphasizing the role of microstructural changes in these areas. An increase in FA in these WM tracts can indicate a compensatory mechanism or a selective vulnerability of different fiber populations in these tracts. Compensatory mechanisms for ALS can involve increased glycolysis, relaxation of synaptic inhibitory events, and faster motor unit firing. Demethylation of the D‐loop region of mitochondrial DNA has been proposed as a compensatory mechanism for mitochondrial DNA (mtDNA) overexpression in carriers of ALS‐linked SOD1 mutations.^98^, ^99^ However, the precise processes of these compensatory mechanisms and their influence on the course of ALS remain unknown.

Another common finding was a correlation between FA values and FC measures within the default mode/hippocampal network,^64^ which reflects the temporal synchronization of neural activity between brain regions, and it involved the medial prefrontal cortex, which is a major node within the default mode/hippocampal network. The correlation between FA and FC in this network can indicate a relationship between the structural and functional integrity of this network, which is involved in cognitive and emotional functions. Two consistent findings were an increase in AD and RD.^30^, ^61^ Increased AD in the hippocampus can indicate a degeneration of the axons, which could lead to neuronal loss and atrophy in this region. Furthermore, increased RD in the hippocampus can indicate a disruption of the myelin sheath around the axons, which could impair signal transmission and synaptic plasticity in this region.

In ALS, the RTOP of water molecules can be used as a biomarker to assess tissue complexity. RTOP reflects the probability of water molecules returning to their original position after diffusion and is sensitive to tissue complexity. This finding was reported by Chen et al.^50^ and involved PhG. The decrease in RTOP in this region can indicate a reduction in tissue heterogeneity and complexity, which could reflect a loss of cellular structures and organization.

Based on fMRI findings, changes in FC, Reho, and ALFF of brain activity in the hippocampal and PhG regions suggest that ALS affects not only motor function but also other cognitive and emotional processes.^47^, ^48^, ^58^, ^64^, ^66^, ^69^, ^72^, ^74^, ^78^ The emotional processing differences that Aho‐Özhan et al.^66^ found suggest that ALS patients can have unique responses to emotional stimuli, which could be related to the observed alterations in hippocampal function and connectivity. Furthermore, the decreased FC in the hippocampal and PhG regions can indicate disrupted neural networks and potential neurodegeneration in ALS patients.^47^, ^48^, ^58^ These results suggest that ALS has a broader impact on brain function beyond motor function. Schulthess et al.^64^ provided further evidence supporting alterations in default mode/hippocampal network connectivity in ALS patients, which could also be linked to cognitive and emotional impairments. Our findings were approved by a recent study that used aberrant multimodal connectivity patterns and found the regional‐node structural–functional connectivity (SC‐FC coupling) of the limbic network (LN)‐related brain regions such as the hippocampus, and PhG was significantly altered.^100^

Interestingly, some studies reported increased FC in certain regions, such as the PhG,^69^, ^72^, ^78^ suggesting that the brain may attempt to compensate for dysfunctional networks by recruiting additional areas. This hypothesis is supported by the findings of Stoppel et al.,^74^ who reported increased novelty‐evoked hippocampal activity across 3 months in ALS patients, potentially reflecting compensatory processes. Finally, these findings imply that ALS patients can recruit additional brain areas to compensate for dysfunctional networks, which may manifest as higher FC and activity in specific brain regions.

ASL is a PWI‐MRI technique that non‐invasively measures CBF in the brain.^101^ ASL has been used to study perfusion changes in neurodegenerative disorders. Based on ASL findings, hypoperfusion in motor‐onset ALS‐FTD was confined to the left PhG, suggesting that this region may be particularly vulnerable in this specific patient group.^42^ This finding could have potential implications for understanding the neuropathological processes that underlie motor‐onset ALS‐FTD and developing targeted therapeutic interventions. Furthermore, significant discrimination between ALS patients and HCs based on CBF in the right hippocampus indicates that alterations in CBF within the right hippocampus could serve as a potential biomarker for the diagnosis of ALS and monitoring disease progression.^45^ Other previous studies in ASL reported disease severity associated with GM and motor neuron involvement, in line with our findings.^102^, ^103^

Kalra^104^ reviews the literature on MRS findings in ALS, focusing on the motor and non‐motor regions affected by the disease. He demonstrated neurochemical changes reflecting neuronal loss or dysfunction NAA is most significant in the motor cortex and corticospinal tracts (CST). Other neurochemical changes observed include increased myo‐inositol (mIns), a putative marker of gliosis. The MRS confirms that the involvement of non‐motor regions such as the frontal lobes, thalamus, basal ganglia, and cingulum is consistent with the multi‐system facet of MND with ALS. In line with our findings, Christidi et al.^40^ found that metabolic alterations in the hippocampal region, specifically tNAA, tNAA/tCr, tCho, Glu/tCr, and inositol, could serve as valuable markers for ALS characterization. Furthermore, certain metabolite associations may be useful for monitoring disease progression and evaluating treatment efficacy.

## 5 LIMITATIONS AND RECOMMENDATIONS

One of the main limitations of this review is the heterogeneity in methodology and patient populations between studies. The studies employed different MRI techniques, scanner strengths, acquisition parameters, and analysis methods, which can introduce variability in the results. The studies also included patients with different ALS subtypes, stages of disease, and genetic mutations, which limits the comparability of findings. Furthermore, some studies had small sample sizes, which can reduce statistical power to detect differences and associations. Another limitation is the cross‐sectional nature of most studies (only four were longitudinal). More longitudinal studies are needed to determine the temporality of changes in the hippocampus and parahippocampal regions relative to clinical changes in ALS. Subfield analysis of the hippocampus can provide valuable insights into the regions affected by ALS and associated with cognitive impairment. Further research should aim to determine specific subfields that can serve as biomarkers for the monitoring and prognosis of the disease. An integrated analysis of multimodal imaging, combining structural and functional MRI with other modalities such as fMRI, DTI, MRS, ASL, and other neuroimaging methods, can yield a more comprehensive understanding of how the hippocampus and parahippocampal regions are affected in ALS. More research on the LATE‐NC characteristics in patients with ALS and ALS/FTD, particularly using MRI. Studies could evaluate whether LATE contributes to the observed hippocampal atrophy and if it correlates with cognitive decline in these patients.

## 6 CONCLUSIONS

The hippocampus and connected medial temporal lobe structures are implicated in memory impairment, functional decline, and disease progression in ALS. Hippocampal atrophy, disrupted connectivity, and altered metabolites correlate with poorer cognitive performance, functional measures, and faster disease progression. The findings highlight those specific hippocampal subregions, such as CA1‐2, dentate gyrus, and fimbria, can be particularly vulnerable. Ultimately, the hippocampus shows potential as a biomarker for disease monitoring, prognosis prediction, and treatment response assessment in ALS. Understanding the relationship between genetic factors, age at symptom onset, cognitive profiles, and hippocampal involvement can provide insights into the heterogeneous mechanisms underlying ALS and its clinical manifestations.

## AUTHOR CONTRIBUTIONS

**Sana Mohammadi**: Conceptualization, Methodology/Study design, Data curation, Writing‐Original draft preparation, Visualization, Investigation, Supervision, Validation, Writing‐Reviewing, and Editing. **Sadegh Ghaderi**: Conceptualization, Methodology/Study design, Data curation, Writing‐Original draft preparation, Visualization, Investigation, Supervision, Validation, Writing‐Reviewing, and Editing. **Farzad Fatehi**: Methodology, Supervision, Validation, Writing‐Reviewing, and Editing.

## CONFLICT OF INTEREST STATEMENT

The authors report no conflicts of interest.

## Data Availability

The data that support the findings of this study are available from the corresponding author upon reasonable request.
